# “No matter what, we just work with the trauma…”: Mental Health Therapists’ Care of Diverse Sexual and Gender Identity Citizens in Cambodia

**DOI:** 10.21203/rs.3.rs-2584144/v1

**Published:** 2023-02-20

**Authors:** Julie E. Bertram, Rachel Kryah, Roxanne Vandermause, Nil Ean, Robert Paul, Adam Carrico, Chhit Sophal, Steven Bruce, Kelly Gregory, Ellen Stein, Julie Mannarino

**Affiliations:** University of Missouri-St. Louis College of Nursing; Missouri Institute of Mental Health; University of Missouri-St. Louis College of Nursing; EMDR Association Cambodia; Missouri Institute of Mental Health; University of Miami; Ministry of Health; University of Missouri–St. Louis; Missouri Institute of Mental Health; University of California, San Francisco; Missouri Institute of Mental Health

## Abstract

The convergence of trauma symptomatology, mental health symptoms, family and social difficulties, and intersectionality of diverse sexual and gender minority (SGM) individual issues is complex, multi-faceted, and challenging for the individuals in Cambodia who suffer them and for the therapists in Cambodia who meet individuals in treatment. We documented and analyzed the perspectives of mental health therapists in the context of a randomized control trial (RCT) intervention within the Mekong Project in Cambodia. The research questions explored perceptions of therapists’ care of mental health clients, therapist wellbeing, and experiences of navigating within a research environment in which SGM citizens with mental health concerns receive treatment. The larger study enrolled 150 Cambodian adults, among which 69 identified as SGM. Three key patterns emerged across our interpretations. Clients seek help when symptoms

interfere with daily life, therapists care for clients and themselves, and integrated research and practice is integral yet sometimes paradoxical. Therapists did not identify differences in terms of how they work with SGM clients compared with non-SGM clients. Future studies are warranted to examine a reciprocal academic-research partnership in which we examine therapists’ work alongside rural community members, evaluate the process of embedding and fortifying peer supports within educational systems, and study the wisdom of traditional and Buddhist healers to address the discrimination and violence that citizens who identify as SGM disproportionately suffer. National Library of Medicine (U.S.). (2020). *Trauma Informed Treatment Algorithms for Novel Outcomes (TITAN)*. Identifier NCT04304378.

## Introduction And Background

Worldwide, mental health disorders are more commonly reported now than in any previous period of human history. Trends from the Global Burden of Disease (GBD) Surveys indicate that the frequencies of depression and anxiety increased by more than 50% and 40%, respectively, between 1990 and 2013 among diverse populations in 195 countries (Vigo et al., 2016). According to the results of the GBD Survey 2019, mental health conditions remain in the top ten for disease burdens (Global Burden of Disease Collaborators, 2022). This trend had remained constant since 1990, but it escalated during the COVID-19 pandemic (Nochaiwong et al., 2021). Since the pandemic, the estimates of the global prevalence of mental health conditions increased by 28.0% for depression, 26.9% for anxiety, 24.1% for post-traumatic stress symptoms, 36.5% for stress, 50.0% for psychological distress, and 27.6% for sleep problems; the variations across geographic regions were partially explained by countries’ different levels of pandemic response preparedness and baseline poverty rates (Nochaiwong et al., 2021).

The financial costs of mental health disabilities are significant, reflected in the direct costs of medications, therapies, and hospitalizations and the indirect costs of lost productivity and income (Trautmann et al., 2016). Low and middle-income countries (LMICs) are disproportionally affected by the burden of these costs, with the populations in these countries accounting for 80% of mental health disorders worldwide (Rathod et al., 2017). Resolving this disparity is one of the most salient challenges to mental health and overall wellness in the modern era, and this is particularly true for Cambodia.

Cambodia is a tropical, southeast-Asian LMIC located in the Indomalayan Realm with a growing population of approximately 17.1 million people (World Population Review, 2022). The mental health needs of the Khmer community compel a caring global response. Anxiety and depression, both considered common mental health conditions, reportedly affect a high percentage of Cambodians, with up to 80% and 53% meeting criteria, respectively (Parry et al., 2020). The prevalence of post-traumatic stress disorder (PTSD) in the Cambodian population has been reported at rates up to 86% (Parry et al., 2020), and substance use disorders are estimated to affect 41.9% of the population, although the true rate is likely higher (Saing et al., 2020).

The high levels of psychological distress in this community are linked directly to distant and more recent exposures to trauma, most notably the agrarian political regime of the Khmer Rouge (1975–1979), which occurred in the aftermath of the Vietnam War. During that period, approximately 1.5 million Cambodian residents died of starvation and sickness, 500,000 individuals were executed, and two million were displaced over the course of several years (Chandler, 1998; Mollica et al., 2014; Tully, 2005).

Political stability was established in the early 1990s after implementation of the United Nations Transitional Authority in Cambodia. However, the country’s violent history set the foundation for transgenerational consequences of trauma. Additionally, the targeting of individuals with advanced education by the Khmer Rouge regime undermined the already fragile health care system, including the mental health institutions and health care clinics responsible for providing direct patient care.

### Limited psychiatric services in Cambodia

As noted, educated individuals were among the groups that the Khmer Rouge regime targeted. The downstream impact of the regime’s political agenda nearly crippled the country’s already vulnerable healthcare and academic research systems. The shortage of medically-trained providers combined with the magnitude of the mental health problems in Cambodia means that almost any psychiatric-centered responses will not meet the needs of the population.

Likewise, the relief provided by nongovernmental organizations (NGOs) is unable to meet the overwhelming challenges. Previous programs initiated by foreign NGOs failed to align funding opportunities and programmed activities according to the long-view needs of the government and academic leadership in Cambodia, resulting in a circumscribed scale-up and inappreciable sustainability. Capacity building is currently underway while Cambodian providers strive to community members’ needs using culturally-derived, traditional approaches.

### Comprehensive mental health programs are needed in Cambodia

In parallel to the lack of therapeutic options, thematically-driven prevention programs focused on mental health are lacking in Cambodia. The historic actions of the Khmer Rouge regime account for a substantial degree of the ongoing anxiety and depression symptoms, but more recent traumatic events also represent a major source of current symptoms. Accordingly, the successful resolution of the mental health care gap in Cambodia mandates attention to be directed toward reducing the frequency and prevalence of trauma-related incidents. Hence, efforts toward preventing psychological trauma should be diverse, multifaceted, and involve varying levels of community, educational, and governmental engagement. Diverse approaches are currently being introduced, but we need data to support the effective integration of such approaches with culturally-responsive care.

One group that suffers trauma-related incidents at significant rates are sexual and gender minority (SGM) individuals. SGM individuals are prone to experience discrimination, intersectional stigma, and violence; mental health challenges are prevalent, affect them more often and with greater intensity than their counterparts (Brody et al., 2016; Brody et al., 2022; Couture et al., 2020; Geibel et al., 2020; Hoefinger & Srun, 2017; Mburu et al., 2019; Saing et al., 2020; Salas & Sorn, 2013; Sopheab et al., 2019; United Nations Development Programme & USAID, 2014; Wieten et al., 2020; Yi et al., 2020; Yi et al., 2018; Yi et al., 2016a; Yi et al., 2016b). In our current focus of research, we sought to understand how an evolving research treatment intervention that oversampled the SGM community was experienced by mental health therapists in Cambodia. This report documents qualitative findings situated within a larger multi-method intervention study.

### Problem and Purpose

The purpose of the present study is to understand the perspectives of mental health care providers in Cambodia who are actively engaged in treatment of Post-traumatic Stress Disorder among SGM.

### Research Questions

1. How do therapists describe their work with their mental health clients?
2. How do therapists describe their own well-being?
3. How do they describe their work within a research environment that involves providing treatment to clients who identify as SGM?

## Materials And Methods

As noted, this qualitative inquiry is part of the larger, ongoing, multi-method (Name of Study) RCT. It is a parallel group RCT where participants are randomized to receive either: 1) three sessions of BA integrated with three sessions of ST (BA + ST) or 2) six sessions of ST alone (ST-Only). Participants complete six individual sessions within the first two months of randomization. Those who continue to display clinically elevated PTSD symptoms at two months (i.e., non-responders) receive Eye Movement Desensitization and Reprocessing (EMDR), an evidence-based exposure treatment for PTSD. All participants (i.e., responders and non-responders) complete a final assessment at four months. The inclusion criteria are as follows: be adults aged 18 or older, male, female, or transgender; have trauma exposure and a score > 31 on the PTSD Checklist (PCL-5); be able to communicate in Khmer; and be able to provide informed consent, which is defined as the ability to accurately paraphrase the purpose of the study. The exclusion criteria are as follows: having active psychosis; a developmental disorder that precludes one’s ability to provide informed consent; and severe alcohol or substance use disorder (other than tobacco) as determined by the Alcohol, Smoking, and Substance Involvement Screening Test (ASSIST).

### Procedures

This qualitative study of the therapists was built upon an established consortium between the Cambodian government and academic researchers in Phnom Penh and the US; focus groups were conducted with stakeholders in Cambodia in 2019. Recruitment for the current study was limited to therapists providing direct care in Cambodia. The purpose of this qualitative arm was to learn from therapists about their experiences working with clients and the research team. We were also interested in their opinions and beliefs about their own well-being. We proposed that therapists’ feedback would help us develop deeper understandings about the larger RCT intervention study.

The informed consent process (Appendix 1) included a discussion of the risks and benefits of participation and the voluntary nature of the research. There were no costs or compensation associated with participation. Participation involved a 1-hour video interview on Zoom, which was recorded and professionally transcribed verbatim. Data on age, gender, education level, profession, and years in the profession were collected from participants. The study protocol and procedures were approved by the US-based university’s institutional review board.

The semi-structured online video interviews were conducted using an interview guide that contained questions pertaining to participants’ experiences working with clients and the research team and their opinions and beliefs about their own well-being. Interview questions were asked uniformly and sequentially. The recorded interviews were conducted in English, as all therapist participants were fluent in the language. The recordings were then transcribed. While it was not necessary to use an English translator with the therapists, we recognized that some passages from the interviews were difficult to discern and some of the transcribed passages were difficult to interpret. To mitigate this challenge, we discussed interpretations as a group and agreed upon the final report of findings.

One methodologist generated fieldnotes for each interview while at least one or more methodologists conducted the interview. Transcripts were deidentified and uploaded onto a secure, password-protected cloud server. Audio and video files were preserved to cross- check the content with the written transcripts.

#### Rigor.

We triangulated data sources, data types, and data collection and analysis methods (Merriam & Tisdell, 2016; Miles et al., 2014). This focus on triangulation allowed us to view all research process phases through a multi-dimensional lens (Merriam & Tisdell, 2016).

### Analysis

The qualitative analysis focused on the results obtained during a one hour interview conducted by internet during the course of the RCT. We developed the analytic plan using two frameworks- focused ethnography and hermeneutic phenomenology. First, focused ethnography was selected because the inquiry is situated within the larger cultural context of the Khmer Rouge genocide that began a multi-generational transmission of trauma in Cambodia. The experience of multi-generational transmission of trauma can be applied to various regions and contexts. As ethnography involves the study of people or culture, its purpose is to locate multi-dimensional understandings of others through a descriptive, interpretive process (Wolcott, 2008). Focused ethnography is defined as a subtype of ethnography in which an area of inquiry is explored within a specific context (Cruz & Higginbottom, 2012).

Second, we selected hermeneutic phenomenology, a methodology with strong philosophical and interpretive traditions, as a lens through which to examine participating therapists’ reflections. Hermeneutic phenomenology is a perspective-taking approach, thinking tradition, and theoretical framework grounded in a philosophy described to study people’s lived experiences (Dibley et al., 2020). The purpose of this method is to facilitate understanding and meaning through a careful listening process and to capture the essence of the phenomena of interest.

At least two members of the research team reviewed the interview transcripts against the audio-visual recordings and made corrections and suggested alternatives for words that were difficult to understand. All transcripts were then reviewed by three analysts. Each researcher independently compared the transcripts with the audio recordings and noted key words, phrases, and ideas that pertained to the research questions and that, in a broader context, described the rich ethnography of the group.

Independent analyses of the interpretations were conducted to delineate patterns in the dataset. The team reviewed all interpretations, looked for areas of common expression and grouped the patterns, identified interpretive texts according to these patterns, thereby covering all interpretive content. Finally, \hermeneutic interpretive descriptions were defined with exemplars from the transcripts.

### Results

#### Demographics.

Four of the five therapists were women and one was a man; two were 25 to 34 and three were 35 to 44 years old. All therapists had a master’s degree in Clinical Psychology and mentored trainees in the delivery of mental health interventions. The mean number of years of professional experience was 9.1, the median was 10 years, and the range was 5−10 years.

##### Findings

Three key patterns emerged across our interpretations of the five interviews as follows:

1. Clients seek help when symptoms interfere with daily life
  - Their suffering is physical and emotional
  - Clients want to be seen and helped, but may hide distress
2. Therapists care for clients and themselves
  - Care for clients is personal, cultural
  - Care for self is necessary
3. Integrated research and practice is integral yet can be paradoxical.

##### Clients seek help when symptoms interfere with daily life

We know from the literature and our experiences in Cambodia that the mental health of the Cambodian people is often challenged by a history of political trauma that is both severe and generational in its effects. It is generally understood that the Cambodian people have suffered due to mass genocide and its sequelae, and that they generally do not freely express mental health issues. These assertions are corroborated by the therapists’ descriptions. Therefore, clients may not present for mental health therapy unless they are experiencing symptoms severe enough to interfere with their everyday lives, rendering them functionally challenged.

According to participating therapists, clients come to treatment with significant trauma histories, as explained by Participant (hereafter P) 3:

…mostly they come with their trauma, especially from domestic violence, sexual abuse, traffic accidents, and problems about relationship and love. Also, some students grow up with their families, especially their parents, not understanding them, not supporting them. A wife comes and tells her husband she has another partner and then they would stay with their family, and fathers use violence against children a lot here. Last year, I worked mostly with victims of sexual abuse. Women who were sexually abused by males or sometime males sexually abused by males, by the priest, by the pastors… It’s what I focus on here, discrimination (Lines 21−29) … No matter what… we just work with the trauma experience (Line 99).

Significant trauma is an important theme because of its universal nature and widespread prevalence. The clients’ suffering is authentic, yet showing up for treatment is considered a sign of great courage and bravery on their part, especially because it is so difficult for them to trust the therapeutic process. Issues as described here, i.e., sexual abuse in any form or infidelity and subsequent guilt in remaining in the relationship with the marital partner, lead to psychological turmoil. Clients had serious dilemmas regarding help-seeking behaviors, as they may have been harmed during help-seeking experiences and/or received conflicting advice about trusting others. Participating therapists worked hard to establish and maintain trust, as the following expert indicates:

They say, “You should not share with other people. You might get hurt or they will use it and threaten you.” “You should not share” is sometimes the teaching here, for example… sharing with other people that you are depressed. There are those who you can trust… but there are many people that no, no, no, you cannot share with, you cannot trust anybody. You cannot trust anybody, so normally they hide it. I can say they hide it, but when they come to us, when they find a psychologist or when we start, everything is very confidential. There is no worry, and then they share with us a lot (P3, Lines 188−193).

Therapists brought up several ways by which an individual’s sharing of their mental health concerns could cause further problems for them, such as being subjected to stigmatization, discrimination, and violence. Several participants explained that generational PTSD and trust issues may be cultural responses to abuses by the Khmer Rouge regime:

I refer to the clients…who are of the generation after the Khmer Rouge regime and their parents. It is more about PTSD, and about trust issues after the Khmer Rouge, and the consequence after the war (P1, Lines 267−272).

In other words, lack of trust is a reflection of the lingering effects of the Khmer Rouge Cambodian genocide that have been transmitted across generations, from parents to their children. As such, therapists strive to build rapport and trust with their clients and help make their clients feel conformable by emphasizing that they are similar to others (e.g., “you’re just the same as any other human being” (P3, Line 206) and that the therapists are here to help. As P3 stated:

Therapists are here to help, but it’s not “I help you”; rather it is we. I say, “we together, we try our best to find a way to deal with the problems or help.”

Thus, clients receive help they need, but at a great cost due to the difficulty seeking it and the stigmatization that can result..

##### Suffering is physical and emotional

Due to the clients’ tendency to avoid discussions of their mental health, they may manifest physical illnesses in response to psychological challenges. Clients communicate awareness through the body that something is painful to them, and they often initially present their experiences and need for help through somatization, as explained by a therapist participant:

…so many people in Cambodia come to us… with many physical symptoms… they complain to us about the physical sensations that they have from their mental problems. So, in our culture, many Cambodian people have problems with sleep, headache, dizziness, [pain] in their bodies, but all those symptoms are related to the emotional symptoms that they have. But mostly they only complain about the physical symptoms (P1, Lines 32−37).

Therapists recognize this somatization:

The way that it’s [explained], that is the way that they express their mental health. It’s … because they cannot sleep. They cannot sleep, so it causes them headaches; it causes them pain, and they have that spinning-in-the- head feeling; it causes them dizziness. They cannot concentrate; they forget a lot, and their moods are swinging up and down. So that’s why they think their bodies are not well-maintained anymore, so they need help. They mostly have to explain [their suffering] through their physical symptoms, and it is hard for them to explain how they feel, how they think (P2, Lines 44−50).

Discrimination and stigma are associated with mental illness due to the idea that a person with a mental health condition is “crazy.” This is described by therapists in the following examples:

I think discrimination and stigma are very important because…the term “mental health” in Cambodia, it means you are crazy… Even now, the people living in the city, they know well about mental health, but most of the people living in the countryside and far away from the city, they still think that mental health [problems] mean you are crazy. It’s also very important, and… if you are crazy, you are discriminated [against] by other people in society (P4, Lines 71−78)

In Cambodia, mental health conditions may also be associated with “a bad spirit” that inhabits an affected person’s body or mind. Clients may turn to fortune tellers or monks for help in overcoming negative emotions:

I think that they believe that all the illness that comes up in their body or their mind might be like some bad spirit or something that they cannot see that is maybe included in the illness. I think that they seek or get treatment from fortune tellers, or from monks, or from anything; I think [that they do this] to overcome their feeling (P4, Lines 94−96).

Although clients who identify as SGM suffer from depression and anxiety at rates similar to those in the general population, some of the causes of their suffering are different. According to a therapist participant, it had more to do with “discrimination, a misbelief in own gender, the expectations of society, the expectations of the family, and the way that society treats them” (P2, Lines 173−175). Transgender issues often revolve around family and society, and clients disclosed to the therapists that the response patterns they have been subjected to include being blamed and being made fun of. “Discrimination from their families especially… And so other people make fun of them and blame them” (P3, Line 29). A therapist participant also noted that clients who identify as LGBT [Lesbian, Gay, Bisexual, Transgender] who sought help [from informal sources] had experienced discrimination from society, family “…and also in religious practice” (P3, Lines 31−32).

Although therapists viewed clients as human beings regardless of their sexual orientation, they said society viewed their clients as “not human” and “not from nature” because “they identified as LGBT” (P3, Lines 35−39). Although Buddhism tolerates the LGBT community, there are religious practices that discriminate against members of that community (P3, Lines 33−34).

The clients who took part in the study who identified as LGBT were often referred to as the “special group” (P2, Lines 160−161), which is a term used throughout the larger community, as a therapist participant explained:

For me there is no difference because they are all true kinds of [people] like us, and they have their own important issues, and they have their own uniqueness. I say that [the group] is special because of society, the way that [society] groups them (P2, Lines 179−181).

Several therapists treated clients for low self-esteem, low self-image, depression, anxiety, and phobia; clients suffer from these symptoms, as one therapist put it, “because society treats them differently because they are in a special group” (P2, Lines 164−166). Relatedly, self-identity concerns were noted by a therapist participant, who stated:

Yes, self-identity [concerns] because, you know, most of my clients are LGBT. They are confused about some of the very difficult decisions they need to make. They don’t know who am I, and they also have relationship problems—love, broken heart…. Also, social influence can cause depression, trauma, anxiety, and PTSD, and back to your question about symptoms, they mostly come with a negative, negative image of self (P4, Lines 26−35).

Some of their clients had also reported having nightmares about trauma or violence and found these repetitive bad dreams distressing, as explained by a therapist participant:

They share things like that they have a lot of bad dreams that repeat what has happened to them in the past. Sometimes there is violence inside those dreams, and they come with nightmares P3, Lines 64−66).

Therapist participants’ clients diagnosed with PTSD often presented with symptoms of anxiety and depression. In the following excerpt, which was reported within the context of the clients’ hopelessness and suicidality, it is apparent that their clients perceived their own suffering from an emotional (e.g., hopelessness, suicidality, fear), physical (e.g., difficulty breathing), and cognitive perspective (perseverative thoughts of doom or similar thoughts) and described their symptoms as highly distressing.

And then other symptoms…this all about a mix between PTSD, anxiety, and depression. They don’t want to live, or they lose hope. They don’t want to stay here and live anymore, and they feel no future and a lot of anxiety from society, and… some clients have breathing difficulties and worry a lot and complain a lot sometimes (P3, Lines 77−80).

Of particular saliency was the theme of the important role that depression plays among individuals diagnosed with PTSD. Other common symptoms among clients who identify as LGBT described as low mood, sleeping difficulty, memory problems, lack of concentration, trouble functioning at work, and difficulty with relationships cut diagnostic categories (P2, Lines 22−25). Therapists recognized the multifaceted nature of the clients’ suffering, who presented with a wide variety of symptoms. Accordingly, therapists faced ambiguity to differentiate among the symptomology, diagnoses, and treatment of their clients.

Participants described dissociation as when a client presents multiple identities during treatment sessions. According to a therapist participant, there are personality factors and cultural manifestations that are hard to distinguish, categorically, from symptoms:

… sometimes, during the sessions… they show dissociation, like from one [identity] to another; they come as one client, and then they leave as another. They say “I’m not quite sure about dissociative identity disorder, but [I know about] split personality.” Because sometimes here [ideas about mental health] mix with the culture… like [that they have] another spirit [inside them]… another spirit does not come inside of them… for us [therapists], we see another personality… a client might come [to an appointment] with another spirit [inside of them] and talk in a different voice, and talk about the supernatural or whatever (P3, Lines 65−70).

This finding is important and culturally specific. Similar accounts have been documented by Hinton et al. (2021), who proposed that auditory hallucinations must be understood within a specific cultural context.

##### Clients want to be seen and helped, but may hide their distress

Therapists indicated that in addition to avoiding seeking help due to discrimination and stigma, clients refrained from disclosing their problems. The combination of a culture of trauma along with the tendency to “hide” mental health symptoms creates a paradoxical situation for clients who need help. Accordingly, they may seek medical (non-mental health) care for relief of physical symptoms, which may involve medication or treatment, reassurance, or other means. However, for their everyday life to be made better, they need to reach out to others for assistance. Thus, the act of seeking care is, in itself, a presentation of suffering that requires courage and deserves a response.

Although shared, complex, multi-generational trauma caused by the political regime of the Khmer Rouge exists in Cambodia, clients do not directly discuss it. They are still struggling to understand how to cope with the painful past. Lack of trust is also a reflection of the war and has been transmitted by parents to their children. Therapists acknowledged that there is a sense that this larger looming trauma (the genocide that took place in the 1970s) affects their clients, but the clients cannot address it directly. Consequently, therapists reportedly spent a lot of time building rapport and trusting relationships with the clients by explaining the research study and providing information about it; this is done in hopes that their clients can become more confident about meeting with them.

Clients are also hesitant to disclose or reveal their problems (“it’s the path of the culture of not talking it out”(P1, Lines 46–52) and their avoidance (“not being able to put the problem on the table” (P1, Lines 46–52) is part of a larger cultural response pattern. For example, a popular Cambodian proverb cautions people to keep problems within the family and not bring them to an outsider, and at the same time to not bring problems from the outside the family into the family. This pervasive silence creates conflicts within those who need help from a therapist. Additionally, clients have limited knowledge of mental health issues, often thinking that therapy is only for the most severe mental disorders, like psychosis and schizophrenia (P1, Lines 63−67).

Despite a general, cultural reluctance to seek professional mental health treatment, clients come to therapy seeking relief from their suffering. However, it is unclear how many Cambodian people suffering from mental health symptoms do not seek treatment. Apparently, only a minority of individuals do seek help, given that treatment seeking usually occurs only when daily life is impaired. As detailed by the therapist participants, prior to seeking therapy, most clients attempted to manage their symptoms on their own by exercising, seeking answers on the internet, through Facebook or other social media platforms, and listening to Buddhist prayers (P1, Lines 101−107).

Some individuals also got involved in charity work or made donations in an attempt to cope with their suffering. A therapist participant further noted that given that clients are coming from a place of discomfort or discordance, although relief is an implicitly understood purpose of treatment, they typically have specific ideas or goals in mind:

They may want to get good advice to help them get off that symptom… they can get medications. They can get a technique; they can get a strategy, a coping strategy to help them because they don’t know why [they are suffering]… but sometimes a small amount [of them]… have higher education or they have knowledge of mental health, so they understand the process of counselling, the process of treatment, so they can Flow with the session. Flow with the full course of the treatment (P2, Lines 74–80).

Historically, a wide range of healing approaches have been practiced in Cambodia to help relieve distress, and as aforementioned, the clients who took part in (Name of Study) tried to manage their own symptoms through physical activity, self-education, and spiritual activity. As such, a therapist participant emphasized the importance of understanding Cambodian cultural practices (e.g., offerings to spirits, fortune telling, and rituals) because they often complement the more Western therapeutic approaches. As explained by the therapist, when families fail to get help from society or the government, they ask the spirit to protect the family, and clients who suffer from numerous traumas also pray and ask for such protection:

…we believe that when someone passes away, their body is still here, but their spirit comes and protects the family and that’s why every day… we prepare food; we do some rituals, and then we ask [for a] kind of help from them, and sometimes we dream about them, and then we are connected… not just my parents [but also] my grandparents, the other generations; they stay together in protecting the whole family (P3, Lines 134−139)

As mentioned earlier, most clients seek therapy expecting to get advice or medication. According to the therapists, those who seek advice are typically looking for ways to help themselves and relieve their symptoms (e.g., “get better sleep and stop thinking about the problems or negative thoughts” P2, Lines 68−74). As described above, treatments were allocated according to protocols as a part of the larger intervention study (National Library of Medicine, 2020).

A therapist participant also noted that self-care practices were disseminated to the population through education campaigns: “we make a video, and then we talk, and then they can learn from it. But at the same time, we say this [is a particular] recommendation from the WHO on how to do self-care” (P3, Lines 190−192). Other recommended and/or prescribed treatments included psychoeducation for clients regarding their symptoms—so that clients can understand their problems and the effects these problems have on their daily live—and working on a treatment plan with a therapist. Therapists also emphasized the importance of family support, as explained in the following excerpt:

And [clients need to] get enough support from therapists, and [we must] make sure that they have support from the family. Mostly, I think that every therapist is important to helping the client, but I personally believe that family and people who are close to them are more important. (P4, Lines 142−156)

##### Therapists care for clients and themselves

The issue of care as a human concern is clear in the therapists’ stories. An exploration of the concept of care is relevant since the storied accounts of these therapists reveal a deep and abiding sense of care-related feelings for the clients. Specifically, how clients were treated is an important issue to these therapists because they were keenly aware of their clients’ suffering and the extremity of what this means regarding the clients’ quality of life. The depth of understanding and care-related feelings that therapists had for their clients, as revealed through their stories, was striking to us. Through this process, we began to see that the communal culture embedded in Cambodian society may be foundational and deep-seated in the practice that connects people and permeates the therapeutic relationships in the country. This phenomenon underscores therapists’ need to focus on their own self-care as well, since they may be exhausted by their fellow citizens’ troubles.

Participating therapists referred to several wellness habits that they practiced regularly, including getting quality sleep, thinking optimistically, getting physical exercise, eating nutritious foods, taking care of plants, and practicing mindfulness meditation, and sharing time, as noted by P2:

I sleep… I am happy… it helps with sleeping… it is really important to me because it helps my brain to work well… also, I eat well, and I exercise almost every day… [I practice] relaxation breathing technique… I enjoy nature. So, nature is all around my house. I really enjoy nature and green environments. I help plant flowers. I enjoy everything like that, and I also connect back with my loved one or with my family (P2, Lines 317−324).

As self-awareness and self-care are important for both clients and therapists, providing therapy is as much about teaching awareness as it is about delivering particular techniques. Therapists recognized that the techniques they suggested to their clients were also useful for their own self-care. For example, a therapist participant referred to STs as an important daily practice, as follows:

For my self-care practice, I sometimes just try to do what I have told to my clients [to do], like always being positive, sleeping well, doing exercise… I also practice my… stabilizers and technique. (P5, Lines 214−217).

One of the therapists also highlighted the importance of having a balanced life: Because I’m also the mother of a son that I need to take care of myself because I have my own [life]… I have the ability to help many people, so … [there’s] all that energy to help my family as well as my son; my family there. It’s balanced, yeah. (P5, Lines 333−335).

Therapists also purposefully employed strategies such as taking small caseloads, taking lunch breaks, driving and enjoying the views, studying philosophy, and meaningful non-work related conversations with others to support their own wellness in their striving for work−life balance.

##### Care for clients is personal, cultural

The therapists provided detailed descriptions about their practice and intimate understanding of the lifestyles and characteristics of the clients they served. They frequently referred to the clients’ emotional needs and to the history of trauma their fellow citizens endured. They also understood the beliefs and health practices, including spiritual practices, that are unique to Cambodian culture. As such, they preferred to meet with clients in the personal spaces where clients described feeling comfortable and experiencing well-being and relief from suffering; therapists also mentioned combining Western and Cambodian cultural treatment practices to achieve optimal outcomes.

It was also evident that the community of therapists was appreciative of the support provided by the US team and was accustomed to the layers of supervision, which provide a foundation for wellness, as noted by P2:

Not only [other therapists] from this project but also from different projects as well… we meet and we talk. We share [details] about a case or some other thing and we support each other. (Lines 268−284).

As a therapist participant explained, sharing among colleagues provided mutual benefits: “We understand that people have to express how they are feeling, so we can express our feelings to one another, and we can [explain things] to each other very well” (P5, Lines 218−219). Although therapists described that personal therapy could also be helpful, actually finding a therapist was difficult because they all knew one another. Their colleagues were all supportive, but that was not the same as having a personal therapist (P5, Lines 331−339).

##### Care for self is necessary

Perhaps because they were so thoughtfully invested in caring for individuals who were suffering and because the culture of their community is indeed communal, therapists could get close to their clients. As a result, they engendered the clients’ trust, understood and empathized with clients. Nonetheless, this can be quite taxing and require therapists to practice self-care continuously. Their descriptions of their self-care and their use of supportive supervision were classic and wholesome reminders to therapists everywhere about the importance of self-care and supportive supervision for both professional and personal life.

According to a therapist participant, altruism and a sense of personal satisfaction derived from helping others contributed to therapist well-being:

I think it’s [good for] my own [well-being]; when other people get better, I feel better. There is like something behind it that makes me not feel tired most of the time because when I see my friends get better, I feel like a butterfly inside. I like to see other people grow in good ways (P3, Lines 377−384)

This therapist also recognized that witnessing the client progress led therapists to experience vitality and inspiration:

These people give me energy… the clients… help me a lot too. They help me feel better and better and enjoy my work when I can see the improvement, or they cooperate well, or I can understand them very well… they have different challenges, so I also learn about how… they can help themselves, how they go through their lives, and things like that. It affects me… but it also helps me to learn more (P3, Lines 311−322).

##### The integration of research and practice is integral yet can be paradoxical

The purpose of the US/Cambodian partnership—to bring the needed mental health care interventions to Cambodia—has provided participating Cambodian therapists with resources they required for treatment and practice, as well as allowed them to master the techniques they may not have been peripherally familiar with. The five therapists we interviewed were grateful for the opportunity to learn. At the same time, their deep understanding of their clients’ well-being needs meant that, at times, they could not prescribe or deliver the breadth of treatment that they would otherwise offer. Some of them felt the need to follow up later to give clients extra help not covered in the (Name of Study) protocol. Though most therapists understood the value of research and the need to stay true to the protocol, it was clear that being a therapist involved in a research protocol caused tensions for some of them; this has important implications for the current research.

In particular, for participant therapists, having to follow protocol meant not disclosing any ill feelings about the tensions of delivering protocol-driven care; however, it was evident that not being free to deliver the type of care they might otherwise have decided upon was a challenge. This is a common research–practice dilemma that is part of the means of reaching the goal of delivering evidence-based treatments. Nonetheless, in this specific case, it might be beneficial to openly discuss these tensions and assess the care delivered in other areas of the country (which is part of the original research plan). Building capacity through dialogic engagement with therapists who are in this state of research−practice tension can propel the project forward.

Therapists recognized that being part of a research study had benefits for both therapists and patients, as the research protocol included BA, ST, and EMDR. Although the five participating therapists had been trained in EMDR prior to the research project, they were unfamiliar with BA (Lines 115−119 and 123). Therapists were comfortable with all the treatments offered, including manualized programs with procedures, the continuity of care, the psycho-education component, and the full scope of ethical care that can be provided as a result of the (Name of Study) project. They were appreciative of the research project and felt that they were helping the clients by treating them ethically and professionally (Lines 335−342). As noted by P2, protocol was always followed during therapy.

Clients can obtain treatment through research studies, but since these studies involve both a treatment and a control group, not everyone will be treated. “We have a procedure, we have a manual, and we have to follow those steps, and we have to follow that manual” (Lines 109−110). Nonetheless, therapists obtain training on the treatment techniques, which can be beneficial for their future work with their clients, as described by P2:

So, we provide behavioral activation, and we connect with them, and we also provide them with stabilization techniques. So, we practice it, and we apply it to the target group, and we see how it works with them and how it helps them to cut down the symptoms to relieve them of their negative activity in their life or thoughts (Lines 91−94).

For therapists who are familiar with some of the techniques used in (Name of Study), there were no major conflicts, as one participant therapist stated:

I think for me it’s not quite [as] difficult because [the techniques] were in the flow, like before, and also… because we already use that here. I was an EMDR therapist before… here we have to be trained professional therapists to be able to treat people. The research project that we joined also used EMDR. Plus, we already provide behavioral activation and stabilization to our clients (P2, Lines 115−119).

Therapists discussed the dialectic of working in a research intervention while at the same time holding an alternate perspective on client needs:

I think for me as a therapist there’s not enough [time] because we spend more time because… you know that I’m a family therapist, that I also have a different lens, a different view for looking at my clients. So, we know that I also spend more time on giving information to the client. [I tell them] “if you need other help, please contact me and if I have time I will also work more …and I provide some treatment and my solution to them. I think this works, yeah (P4, Lines 205−211).

According to a participating family therapist who has undergone training on crisis intervention, the BA works, but therapists may also have other ideas, depending on the symptoms the client presents and the therapist’s assessment:

It depends on the client. If we assess that the client needs urgent help, I include it during the therapy session as well because normally we spend like 14 minutes or 15 minutes for one session, and if we know that the client needs urgent help, maybe I spend more than an hour to help them. If we assess that the client is fine, then we just give them good information: “if you need other help, please contact us,” something like that. Like, extra help (P4, Lines 222−225).

One therapist participant also noted that the randomization process was helping clients get the kind of treatment they needed about 50% of the time:

Yes. It depends. Because it might be helpful when we know… we choose the randomization when we have already chosen the technique, when a treatment plan has already been fitted to every client, something like that, right? I think it might be helpful, but it depends. Maybe the client does not need that process, and sometimes they need another process. And also, I think they learn that it [a procedure] might be helpful, if not 100% [of the time] and maybe the client sometimes needs more than that or another technique. For me, I think it should be 50% (P5, Lines 228−234).

When therapists provide clients with treatment as a part of a research protocol, there are subtle aspects of the experience that conflict with the usual practice of these therapists. For instance, a therapist participant mentioned having the “urge to do something” beyond the research protocol treatment (P1, Lines 134−136). This therapist further indicated that they have followed the research protocol and were also gathering information that will be important in the future.

P2 acknowledged the potential for conflict, but was also of a view that it was a “…small thing. Yeah, sometimes, but it depends on the client. They need to be flexible but sometimes because we have to flow with the research” (Lines 132−133). Although the study was working for the therapist, and the protocols were clear, the therapist still expressed a hint of conflict: “I really appreciate the work that we do for people in the project and even if we have to follow the procedure, at least we can still help them” (Lines 335−337).

It is also noteworthy that, according to P3, some therapists used STs before engaging in the research project and found that these techniques reduced PTSD symptoms among their clients. P3 did not know a lot about BA prior to the study, but indicated that it was a good combination for their clients, in particular the self-care component (P3, Lines 234−240) because it reduced the severity and frequency of PTSD symptoms (P3, Line 278). However, therapists were still of a view that they needed multiple approaches to help their clients, as explained in the following excerpt:

I think I mix some [treatments] for my clients for doing self-care [after a session or other activity]. I mixed [treatment types], but at the time I did not know [that doing thus was a technique]; I did not intend to do that. But now when we do (Name of Study), we use a mix between behavioral activations and stabilizations. So, for me, it’s good. It is not just the therapy in the sessions but also the BA that follows at home. For example, it is important to decide how many [treatments], and then talking and walking or something like that (P3, Lines 244−246).

Actually, to be honest, I think the best way is a mix between BA and stabilizations. That means they still get treatment, but now they also get screened because when we ask them to [wait for screening], for example, it’s not really good for them because normally here… as I shared with you, they hide [their feelings] inside. And when they suddenly share with us, they say they are doing much better. Sometime the clients go [from one place] to another place because they normally feel that no one cares. They just act like that. However, the stabilizations mixed with the BA is just symptom reduction. It’s just symptom reduction (P3, Lines 280−283).

## Discussion

The convergence of trauma symptomatology, mental health symptoms, family and social difficulties, and issues is complex, multi-faceted, and challenging for the individuals in Cambodia who suffer them and for the therapists in Cambodia who meet individuals in treatment. A concept that has been used in the literature to convey this complexity is “syndemic,” meaning that there are multiple factors working together to create heightened risk for disease (Couture et al., 2020). Understanding syndemic frameworks can be helpful for communities as they seek to address populations at risk for various complex disease processes.

The language clients used to communicate suffering matters and understanding it helps therapists better understand their clients’ needs. Therapists in Cambodia are able to speak the language of their clients’ suffering because they share a cultural past with their clients, understand the expressions and idioms of distress, and are poised to deliver culturally sensitive, relationship-grounded care.

When therapists understand and make meaning of the suffering their clients experience, both parties attain benefits from the therapeutic process and move forward despite the burden of various symptoms. The participating therapists conveyed their empathy—the ability to have an intimate understanding of the other (Galetz, 2019)— through their rich descriptions of self-care and their thorough comprehension of the issues their clients experienced. The dialectic of self-care as an essential aspect of care for the other reportedly required a tenuous balancing, but was beautifully demonstrated by this group who learned about therapeutic excellence through practice. This empathic ingenuity among therapists is a testimony of the recovery, hope, and exquisite humanity—which was conveyed through care and allyship—of this dedicated, committed group of professionals. This tapestry of soulful and informed practice prompted us to ponder ways we could learn from these wise therapists about tender care of the self, combined with principled care of clients, excellent commitment to the supervision process, and a mindset of growth toward an integrated, supportive network and the lifting up of all citizens.

The research–practice paradox and the resulting tensions described in the findings are not new. In this study, we learned that the therapists understood the principles of research and the need to follow protocols in implementing interventions. However, they also observed that this caused them some discomfort by limiting their ability to consider the often nuanced cultural needs of the clients when planning interventions. In other words, had they not been holding to research protocols, they might have expanded their interventions based on client needs to include procedures that were not included in the treatment protocol. Although they appreciated learning or practicing the known interventions, they did not have the freedom to practice outside of the scope of the study. This paradox exists in research partnerships where the roles of the researcher and practitioner overlap (Ledger, 2010). It is an issue to be considered in all such projects.

Nonetheless, approaches that include research participants in the evaluation of interventions hold promise for ensuring that the interventions are delivered according to the needs of local communities. Community-based participatory approaches challenge us to collaborate at all stages: from the inception of the study and the drafting of the research questions to the development of interventions, gathering of the data in ethically sound ways, analysis, and reporting of the findings in ways that help community members move toward their stated goals. Future research practice projects that qualitatively explore how to include evidence-based interventions alongside complementary or sequential cultural interventions could also be developed.

As researchers, we attempted to stay true to the methods that we selected to carry out the research project. For this study, we applied focused ethnography and hermeneutic phenomenology strategies during data collection and analysis. As a research team, we collected data together, generated field notes, discussed interpretations during team meetings, and reviewed the findings for plausibility.

This study provided us with important insights about engaging in an international research collaboration. First, developing nations such as Cambodia have a wealth of cultural beliefs, traditions, and practices that provide them with ways to navigate through complex social situations like multi-generational trauma, poverty, discrimination, and stigma. Thus, to more fully understand these experiences, we listened to and learned from the Cambodian therapists, offering them ample opportunities to share what they know with wider audiences.

Second, our results inspired us to consider new questions and possibilities. How could we empower citizens and therapists to develop telehealth resources (e.g., infrastructure and Wi-Fi capabilities) and provide citizen training on how to use these tools? How might therapists connect with rural community members who identify as SGM and need support and accurate information about mental health and symptom management; research corroborates that telehealth improves access to mental health treatment in resource-constricted regions (Acharibasam & Wynn, 2018). Another promising approach in this context of listening and learning from therapists is to embed the support of peers who identify as SGM within education systems. As these individuals often have lived experiences recovering from mental health conditions, substance use disorders, or both, they can provide education and practical support to others who are living with these issues while they attend school (Worrell et al., 2022).

Finally, the wisdom of traditional and Buddhist healers has long been relied upon to support and provide a sense of meaning to the citizens of Cambodia. These local healers offer a non-Western, non-medical perspective on life and wisdom on existential matters that may be particularly helpful for Cambodians as they reconcile with their painful past, navigate their present circumstances, and look toward future directions in living (Eisenbruch, 2019). Future studies are warranted to examine a reciprocal academic-research partnership in which we examine therapists’ efforts to integrate various psychotherapeutic approaches with cultural traditions, evaluate the process of embedding and fortifying peer supports within rural and educational systems, and study the wisdom of traditional and Buddhist healers to address the discrimination and violence that citizens who identify as SGM disproportionately suffer.

## Data Availability

The datasets used and/or analysed during the current study are available from the corresponding author on reasonable request and following execution of a Data Transfer Agreement.
